# Systematic Review of the Effects of Phosphodiesterase-5 Inhibitors and Dexamethasone on High Altitude Pulmonary Edema (HAPE)

**DOI:** 10.51894/001c.7111

**Published:** 2019-03-04

**Authors:** Amy Bliss, Sonia Mahajan, Kevin M. Boehm

**Affiliations:** 1 Department of Pediatrics, Division of Pediatric Emergency Medicine University of Utah. Salt Lake City, UT; 2 Department of Emergency Medicine Sacramento Medical Center. Sacramento, CA; 3 Michigan State University College of Osteopathic Medicine, Department of Osteopathic Medical Specialties; Broward Health. Fort Lauderdale, FL Department Emergency Medicine

**Keywords:** dexamethasone, phosphodiesterase-5 inhibitors, high altitude pulmonary edema, acute mountain sickness

## Abstract

**OBJECTIVE:**

To review and synthesize the current available evidence of the effects of phosphodiesterase-5 inhibitors and dexamethasone on the outcomes of individuals affected by acute mountain sickness symptoms and High Altitude Pulmonary Edema (HAPE).

**METHODS:**

In 2015, two authors independently performed separate searches using three different databases (PubMed, Ovid and Web of Science) later reviewed by the third author. The searches used the following terms “High Altitude Pulmonary Edema” and “Phosphodiesterase-5 Inhibitors” while the second search used “High Altitude Pulmonary Edema” and “Dexamethasone”. The following exclusion criteria were utilized: patients < 18 years old, non-human studies, studies at altitudes < 2,000 meters. The search included articles from year 2000 to current.

**RESULTS:**

A total of 237 manuscripts were initially reviewed. The search involving phosphodiesterase-5 inhibitors initially yielded 37 manuscripts, four of which met inclusion criteria. A total of 101 patients were included in these articles. For the Dexamethasone search, 200 manuscripts were retrieved. Three of these studies met the inclusion criteria, reporting data on a total of 66 patients. None of the studies reported significant improvements in outcomes of patients from the use of either phosphodiesterase-5 inhibitors or dexamethasone.

**CONCLUSIONS:**

According to the current available literature, neither phosphodiesterase -5 inhibitors or dexamethasone significantly alter the outcome of individuals affected by HAPE.

## INTRODUCTION

Areas with high altitude are becoming more and more common as destinations for people traveling for business and/or pleasure. High altitude pulmonary edema (HAPE) is a potentially life-threatening, non-cardiac, pulmonary edema that affects otherwise healthy individuals at high elevations; specifically altitudes of 2,000 meters and greater.^1^ The prevalence of altitude sickness, more specifically Acute Mountain Sickness (AMS) has relatively recently been observed at levels as high as 36.7%^2^ and 34.0%.^3^ On average, about 40 million people travel to elevations in the US that put them at risk for developing different AMS symptoms along the high altitude sickness spectrum, including HAPE.^4^ In addition, an increasing number of people are traveling to elevations greater than 4000 meters around the world.^4^ Consequently physicians, specifically emergency medicine physicians, may encounter any part of the spectrum of AMS conditions with increasing frequency.

HAPE is at the more severe end of the altitude illness spectrum and the leading cause of death from altitude illness.^5^ It is a non-cardiogenic pulmonary edema with a multi-factorial pathophysiology with pulmonary hypertension at the cornerstone of its mechanism.^4^ Auerbach described the typical cascade of HAPE as follows: The higher a person ascends up a mountain, there is a lower arterial partial pressure of oxygen. This causes hypoxic pulmonary vasoconstriction that will cause an increase in pulmonary hypertension. This results in over perfusion of the lungs that causes a vicious cycle of pulmonary and peripheral venous constriction that in turn causes an increase in pulmonary blood volume. As this continues, there is an increase in capillary pressure that will eventually cause capillary leak, thus decreasing alveolar sodium and water clearance, resulting in HAPE.^4^

The management of HAPE is aimed at both prevention and treatment. Prevention involves acclimatization and controlled ascent, which helps to maintain consistent oxygen delivery to tissues.^6^ Additionally, acetazolamide, a carbonic anhydrase inhibitor, has been used to help prevent HAPE. The gold standard treatment for HAPE is rapid descent. Not every situation permits rapid descent, however, so other options for treatments include oxygen supplementation and pharmacotherapy. This review focuses on two medications in particular. The first medication, dexamethasone, stimulates alveolar sodium and water reabsorption and enhances nitric oxide availability in pulmonary vessels.^7^ The second class of medications are phosphodiesterase-5 inhibitors, which enhances pulmonary vasodilation.^8^ The purpose of this paper is to review and synthesize the current available evidence of the effects of these two medications on HAPE and AMS symptoms.

## MATERIALS AND METHODS

The first two authors independently searched three different databases: PubMed, Ovid Medline and Web of Science. The first author used the following terms “High Altitude Pulmonary Edema” and “Phosphodiesterase-5 Inhibitors” in each of the databases. The second author searched for results using the following, “High Altitude Pulmonary Edema” and “Dexamethasone” in the same databases. The results of the searches were reviewed by both of the authors and later reviewed by the third author. The authors then reviewed the title, abstract, and full-text reviews and abstracted data from the studies.

The following exclusion criteria were utilized: patient < 18 years old, non-human studies, altitudes < 2,000 meters (m) studies. The search included articles from year 2000 to current as there were no reports of HAPE and Phosphodiesterase-5 Inhibitors prior to 2000. Only randomized controlled trials that reported human data on the effects of these two medications were included. These exclusion criteria were selected to ensure only adult, human studies were analyzed in our study as HAPE does not occur in altitudes of under 2,000 m.

The same two authors independently reviewed the eligible studies and extracted data on study objectives, number of subjects, interventions, comparisons, and relevant outcomes. The third author reviewed these findings. The results of each study were examined and compiled into tables.

## RESULTS

The search terms initially yielded a total of 237 manuscripts were retrieved initially with all search terms. The “phosphodiesterase-5 inhibitors” and “high altitude pulmonary edema” search initially yielded 37 manuscripts, four of which met inclusion criteria and were randomized controlled trials. Two of the six papers examined the effects phosphodiesterase-5 inhibitors as well as dexamethasone. A total of N = 101 patients were included across these articles. The search using “dexamethasone” and “high altitude pulmonary edema,” initially generated 200 titles. Three of these studies met the inclusion criteria, reporting data on N = 66 subjects (Figure 1).

**Figure:**
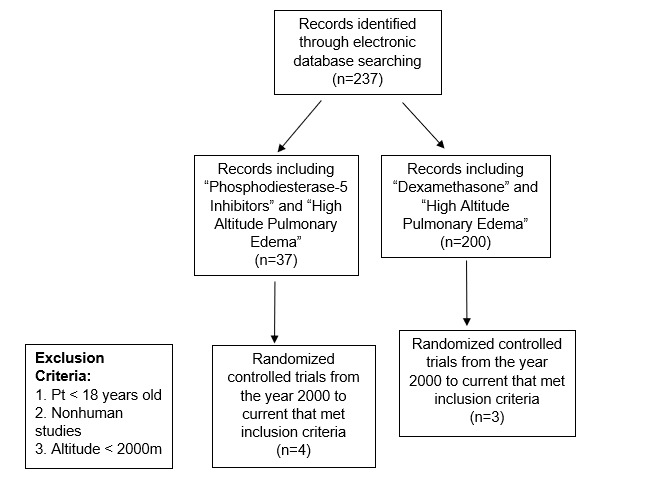
Figure 1 Article Eligibility Criteria

### Summary of Sildenafil Studies

The first 2006 study reviewed was conducted by Hsu et al.^8^ This study examined the effects of 5-phosphodiesterase (5-PDE) inhibitor, sildenafil, during normoxic (i.e., normal oxygen concentration) exercise and during exercise at simulated high altitude conditions causing hypoxic exercise. The study involved 11 healthy, non-smoking male cyclists and triathletes ages 18-35 who volunteered for the study; one withdrew during testing. A total of 10 men performed one practice and three experimental trials at sea level and simulated high altitude of 3,874 m (subjects breathed 12.8% oxygen). Double-blinded capsules (placebo, 50mg sildenafil, or 100mg sildenafil) were given one hour before exercise. For the high altitude trials, subjects began breathing the hypoxic gas for one hour prior to exercise.

Cardiovascular and performance variables measured included oxygen consumption, respiratory exchange ratio, cardiac output, stroke volume, heart rate, oxygen saturation (SaO2), systolic blood pressure, and perceived exertion. They found that sildenafil had no effects on any cardiovascular or performance measures had no effects while at high altitude, although sildenafil increased stroke volume, cardiac output and SaO2. No dose response effects were observed. A post hoc analysis compared sildenafil responders versus non-responders. This study found that sildenafil can significantly improve cardiovascular function while cycling in an acute hypoxic environment.^8^

The second study reviewed was completed in 2005 by Ricart, et al.^9^ It examined the effects of the 5-phosphodiesterase (5-PDE) inhibitor, sildenafil, on pulmonary arterial pressure as well as oxygen transport and cardiopulmonary parameters in humans during exposure to hypobaric hypoxia at rest and after exercise. In this double-blind study, 100 mg sildenafil or placebo was administered orally to 14 healthy volunteers 45 minutes before exposure to simulated altitude of 5,000 m. Arterial oxygen saturation, heart rate, tidal volume, respiratory rate, left ventricular ejection fraction, and pulmonary arterial pressure were measured first at rest in normoxia, at rest and immediately after exercise during hypoxia, and after exercise in normoxia. Measurements of the effect of sildenafil on exercise capacity during hypoxia did not provide conclusive data, although it was noted that sildenafil diminished pulmonary hypertension induced by exposure to hypobaric hypoxia at rest and after exertion.^9^

An additional 2009 study by Lalande et al. set out to determine the effects of acetazolamide and sildenafil on ventilatory control and breathing efficiency during submaximal steady-state hypoxic exercise in a sample of 15 healthy individuals. Following 18 hours of hypoxic exposure in an altitude tent at an oxygen concentration of 12.5% (simulated altitude of 4,300 m), participants performed 10 minutes of hypoxic exercise on a stationary bicycle at 40% of their sea level peak oxygen uptake (VO2) while randomly receiving sildenafil 40 mg, acetazolamide 125 mg, or a placebo. There was no difference in VO2 during exercise between conditions while subgroup SaO2 levels were greater with acetazolamide compared to both placebo and sildenafil. Acetazolamide increased ventilation and reduced end tidal carbon dioxide (CO2) compared to placebo and sildenafil. Breathing was less efficient with acetazolamide in comparison to placebo and sildenafil, while sildenafil did not change VE/VCO2 during hypoxic exercise. Specifically, researchers found that sildenafil decreases pulmonary hypertension when subjects are exposed to acute hypoxia.^10^

The final 2011 study involving phosphodiesterase-5 inhibitors that was reviewed was performed by Bates el al.^11^ This study examined the effect of chronic sildenafil administration on pulmonary artery systolic pressure and symptoms of AMS during acclimatization to high altitude. Sixty-two healthy volunteers were flown to La Paz, Bolivia (3,650 m), and after four-to-five days of acclimatization, they ascended over 90 minutes to 5,200 m. The treatment group (N = 20) received 50 mg sildenafil citrate three times daily. Pulmonary artery systolic pressure (PASP) was recorded by echocardiography at sea level and within six hours, three days, and one week at 5,200 m. There was no significant difference in PASP at 5,200 m between sildenafil and placebo groups. Sildenafil administration did not affect pulmonary artery systolic pressure in healthy lowland subjects at 5200 m but AMS symptoms were significantly more severe on Day 2 at 5,200 m with sildenafil. Ultimately, the data examined in this paper did not support the prophylactic use of sildenafil.^11^

**Table:** Table 1 Summary of Sildenafil Articles Reviewed

**Study**	**N**	**Patients**	**Intervention**	**Comparison**	**Relevant Outcomes**
Hsu et al. (2006)	10	Healthy, non-smoking male cyclists and triathletes	Sildenafil	At sea level vs simulated high altitude	At high altitude, sildenafil increased stroke volume, cardiac output and SaO2
Ricard et al. (2005)	14	Healthy males who normally live at sea level	Sildenafil	Hypobaric hypoxia at rest and after exercise	Sildenafil diminishes pulmonary hypertension induced acute exposure to hypobaric hypoxia at rest and after exercise
Lalande et al. (2009)	15	Healthy males and females	Sildenafil	Sildenafil vs acetazolamide vs placebo all at simulated altitude of 4,300 m	Sildenafil did not affect breathing efficiency
Bates et al. (2011)	62	Healthy males and females	Sildenafil	The difference in pulmonary artery systolic pressure at high altitude with sildenafil vs placebo	There was no significant difference in pulmonary artery systolic pressure at high altitude between the sildenafil and placebo groups

### Summary of Dexamethasone Studies

In the search utilizing the terms “dexamethasone” and “high altitude pulmonary edema”, the first 2006 study by Maggiorini et al.^7^ compared dexamethasone and tadalafil with placebo to ascertain reduction in the incidence of HAPE and AMS symptoms in those adults with a history of HAPE. This was a randomized, double blind, placebo controlled study with 29 patients. Patients were randomized to receive prophylactic tadalafil (10 mg), dexamethasone (8 mg), or placebo twice a day during ascent and stay at 4,559 m. Ascent was from 490 m within 24 hours and the stay at 4,559 m was for two days. HAPE was diagnosed with chest x-ray findings (score > 1/infiltrate or alveolar edema in one or more lung fields) and the presence of AMS was defined as a Lake Louise Score > 4.

Doppler echo was used to measure systolic artery pressure and nasal potentials were measured as a surrogate marker of alveolar sodium transport. Two participants who received tadalafil developed severe AMS symptoms on arrival at 4,559 m and withdrew from the study (no signs of HAPE at this time). HAPE developed in seven of nine participants in the placebo subgroup, one in eight in the tadalafil subgroup, but none in the dexamethasone subgroup. Systolic pulmonary artery pressure was increased less in those receiving dexamethasone and tadalafil, than those on placebo. This showed improved response to dexamethasone for HAPE treatment.^7^

The second 2009 study reviewed was conducted by Fischler et al.^12^ This study examined 23 subjects with previous HAPE, randomized to receive Dexamethasone 8 mg twice daily, Tadalfil 10mg twice daily, and placebo prior to ascent. Baseline cardiopulmonary exercise test (CPET) and echo were performed at 490 m, two-to-four weeks before ascent to 4,559 m. Subjects were taken by cable car from 1,100 m to 3,200 m, from where they continued by foot for approximately 1.5 hours until they reached 3,650 m. After an overnight stay, the study participants climbed under professional guidance within four-to-five hours to 4,559 m, where CPET was performed four-six hours after arrival and echo was performed on the following day.

The study’s results indicated that compared with placebo, dexamethasone improved maximum oxygen uptake, oxygen kinetics, and reduced the ventilator equivalent for CO2. Dexamethasone improved exercise capacity, oxygen uptake kinetics and limited hypoxia-induced pulmonary hypertension at 4,559 m in HAPE-susceptible individuals, whereas tadalafil did not significantly improve exercise capacity and somewhat less-limited hypoxia-induced pulmonary hypertension. Peak oxygen saturation did not differ significantly between the three study subgroups. Pressure gradient over TV (indirect measure of PA pressure) was significantly less for both dexamethasone and tadalafil compared to placebo.

AMS levels improved significantly in the dexamethasone group. Peak exercise capacity decreased in all groups however with the smallest decrease in the dexamethasone group. Overall, dexamethasone was shown to be superior in controlling symptoms and incidence of HAPE compared to both placebo and tadalafil.^12^

This was further assessed by Siebenmann et al.,^13^ who extended the study design to include up to five days. Twenty-four subjects with previous HAPE exposure were included. They traveled to 1,205 m by cable car then continued by foot to 3,647 m where they arrived in the late afternoon and spent the night. The next morning, they made their ascent to 4,559 m. They stayed there for five days. All these individuals were evaluated on bicycle ergometers at an altitude of 490 m (two-three weeks before) and at 24 hours after rapid ascent to 4,559 m.

Maximal workload, heart rate, minute ventilation, calculated maximal voluntary ventilation, respiratory frequency, tidal volume, respiratory exchange ratio, and arterial oxygen saturation were measured. Results indicated that at 4,559 m, maximal oxygen uptake was higher in the dexamethasone group compared to control. Dexamethasone reduced the hypoxia related decline in maximum oxygen uptake. Dexamethasone also reduced AMS symptoms compared to control group patients.^13^

**Table:** Table 2 Summary of Dexamethasone Articles Reviewed

**Study**	**N**	**Patients**	**Intervention**	**Comparison**	**Relavent Outcomes**
Maggiorini et al. (2006)	29	Adults with previous HAPE	Tadalafil and Dexamethasone	Tadalafil vs dexamethasone vs placebo to ascertain reduced incidence of HAPE and AMS in those with previous hx of HAPE	Both dexamethasone and tadalafil decrease systolic pulmonary artery pressure and may reduce incidence of HAPE. Dexamethasone also helps to reduce incidence of AMS in these individuals
Fischler et al. (2009)	23	Subjects with hx of previous HAPE	Tadalafil and Dexamethasone	Dex vs tadalafil for improving exercise capacity (by reducing hypoxia induced pulmonary vasoconstriction)	Dexamethasone may improve exercise capacity during hypoxia in HAPE-susceptible mountaineers
Siebenmann et al. (2011)	24	HAPE susceptible individuals	Dexamethasone	Placebo vs dexamethasone for maximal oxygen uptake at high altitudes	Dexamethasone prophylaxis increase maximal oxygen update (generally reduced due to hypoxia) of HAPE-susceptible individuals for prolonged period of time, without affecting arterial oxygen saturation at maximal exercise

## DISCUSSION/LIMITATIONS

When considering the proposed mechanism for HAPE, immediate descent appears to be the best treatment of choice. By decreasing altitude, there will be an increase in the percentage of oxygen available, thus increasing the PaO2, decreasing the altitude hypoxia, and changing the rest of the negative cascade that follows. The phosphodiesterase-5 inhibitors reduce pulmonary and peripheral venous constriction thus countering the increase in capillary pressures that occur on the cascade, thus complete preventing HAPE. Dexamethasone works on the portion of the cascade that causes capillary leak, increasing the sodium in the alveoli and increasing water reabsorption further down the cascade.^4^

This systematic review examined the current available literature on the effects of phosphdiesterase-5 inhibitors and dexamethasone on physiologic variables associated with HAPE. Overall, the studies involving phosphodiesterase-5 inhibitors had small sample sizes. Hsu et al.^8^ only had a sample size of ten while Bates et al^11^ examined the largest number of subjects with a sample size of 62. Each study measured different physiologic variables including but not limited to pulmonary artery pressure, stroke volume, cardiac output, ventilatory control, breathing efficiency, and arterial oxygen saturation. Additionally, three of the studies simulated high altitude and/or hypoxia while only one tested subjects in the field at an actual high altitude.^8–11^

Also, both simulated and actual elevations differed from 4,300 m to 5,200 m, a difference of almost 1,000 m. The effects of two different phosphodiesterase-5 inhibitors were investigated in the different papers: sildenafil and tadalafil. The differences in variables examined and methods create difficulty in comparing the results of these studies. Overall, the studies included in the review that examined phosphodiesterase-5 inhibitors did show some changes in physiologic variables but the overall impact of these medications on outcomes of individuals with HAPE was not examined.

After reviewing the results of these studies, it may be postulated that, given the mechanism of action of phosphodiesterase-5 inhibitors, it could improve outcomes in patients with HAPE. However, there is no current conclusive evidence that this is correct. Additionally, there are many unknown factors pertaining to each individual patient, specifically pharmacogenomics. The authors believe that the challenges of further research would be difficult since HAPE is so multifactorial as already indicated and environmental in nature.

To better study HAPE, one suggestion would be to conduct a larger randomized trial at a fixed location (e.g., base camp at Everest) for a longer period of time to separately test the effects of these types of medications. With the popularity of mountaineering increasing, we believe that there will likely be more opportunities for studies in base camps around the world. Additionally, there will be more diversity within the subjects included in analytic samples.

Three papers which examined the use of dexamethasone and its effects on high altitude pulmonary edema were reviewed for this systematic review. Unfortunately, the sample sizes in each of these papers were small, ranging from 23 to 29. Two of the papers compared dexamethasone to tadalafil ^7,12^ while only one examined dexamethasone alone.^13^ Again, these papers examined different physiologic variables including pulmonary artery pressure, VO2 max, and oxygen saturation, making comparison of data difficult. In general, the studies involving dexamethasone that were reviewed demonstrated improved physiology at high altitude when compared to a placebo.

## CONCLUSIONS

Each year, there has been an increasing number of people traveling to high altitudes.^4^ Although there is an exhilarating thrill behind such forms of recreation, there is also a risk of exposing self to various forms of AMS and HAPE. On the extreme end of this AMS illness spectrum is HAPE, which can prove life threatening. In an ideal situation, the best treatment option appears to be immediate descent. However, in certain circumstances, this may not be a feasible immediate option.

Alternately, the use of pharmacotherapy to allay AMS and HAPE symptoms until descent is possible offers temporizing methods to decrease significant morbidity and mortality. Although our review of the literature in this area provides some insight on available treatment options, further studies examining the specific effects of phosphodiesterase-5-inhibitors and dexamethasone are warranted.

### Conflict of Interest

The authors declare no conflict of interest.
